# Sural Nerve Perfusion in Mice

**DOI:** 10.3389/fnins.2020.579373

**Published:** 2020-12-10

**Authors:** Anete Dudele, Peter Mondrup Rasmussen, Leif Østergaard

**Affiliations:** ^1^Center of Functionally Integrative Neuroscience (CFIN), Department of Clinical Medicine, Aarhus University, Aarhus, Denmark; ^2^The International Diabetic Neuropathy Consortium, Aarhus University Hospital, Aarhus, Denmark; ^3^Department of Neuroradiology, Aarhus University Hospital, Aarhus, Denmark

**Keywords:** mice, sural nerve, nerve blood flow, hindlimb temperature, red blood cell velocity, two-photon microscopy

## Abstract

Peripheral nerve function is metabolically demanding and nerve energy failure has been implicated in the onset and development of diabetic peripheral neuropathy and neuropathic pain conditions. Distal peripheral nerve oxygen supply relies on the distribution of red blood cells (RBCs) in just a few, nearby capillary-sized vessels and is therefore technically challenging to characterize. We developed an approach to characterize distal sural nerve hemodynamics in anesthetized, adult male mice using *in vivo* two-photon laser scanning microscopy. Our results show that RBC velocities in mouse sural nerve vessels are higher than those previously measured in mouse brain, and are sensitive to hindlimb temperatures. Nerve blood flow, measured as RBC flux, however, was similar to that of mouse brain and unaffected by local temperature. Power spectral density analysis of fluctuations in RBC velocities over short time intervals suggest that the technique is sufficiently sensitive and robust to detect subtle flow oscillations over time scales from 0.1 to tens of seconds. We conclude that *in vivo* two-photon laser scanning microscopy provides a suitable approach to study peripheral nerve hemodynamics in mice, and that local temperature control is important during such measurements.

## Introduction

Nerve blood flow is important for peripheral nerve function and nerve blood flow deficits have been implicated in peripheral nerve pathologies, e.g., diabetic peripheral neuropathy (DPN) and neuropathic pain conditions (Cameron and Cotter, 1997; Lim et al., 2015). Metabolic demands of neural tissue are very high and this is reflected in the tissue blood flow. Peripheral nerve blood flow is comparable to blood flow measured in cortical and spinal cord gray matter, and is higher than in white matter of the spinal cord (Zochodne, 2018). However, unlike the brain, peripheral nervous tissue is not prone to ischemic injury, partly due to its redundant, segmental blood supply and high overall ischemic tolerance. It takes as long as 1–3 h of ischemia to induce permanent axonal damage in peripheral nerves (Zochodne, 2018). However, peripheral nerves affected by diabetes are more sensitive to ischemic damage and this has been attributed to chronic insufficient oxygenation due to capillary dysfunction (Østergaard et al., 2015).

The measurement of blood supply in peripheral nerves is challenging, particularly at the capillary level. Although chronic hypoxia has been implicated in the onset and progression of DPN, nerve blood supply has thus far mainly been measured in the larger, more proximal peripheral nerves (Tuck et al., 1984; Newrick et al., 1986; Østergaard et al., 2015). In the larger nerves, such as the rat sciatic nerve, absolute nerve blood supply (in mL blood per 100 g tissue per minute) can be measured by radiotracer and hydrogen clearance methods, while relative nerve blood flow can be estimated over time using laser Doppler flow (LDF) probes. LDF measurements, however, have limited spatial specificity as they are based on laser light reflected by moving red blood cells (RBC) down to depth of 250 μm, making this method prone to signal contributions from surrounding tissue, particularly in small nerves (Zochodne, 2018).

Accurate measurements of blood flow in small distal, peripheral nerves in the 50–200 μm diameter range are important, not only to understand how their metabolic demands are met, but also to understand whether limited oxygenation and microvascular changes play a role in the pathophysiology of diseases that first affect the most peripheral nerve fibers, such as DPN (Gonçalves et al., 2017). Such measurements are extremely challenging, however, as these nerves receive their blood supply from just a few microvessels, often on the nerve’s surface, as nerve diameters approach the typical diffusion lengths for molecular oxygen.

The aim of this study was to develop an approach to characterize distal peripheral nerve hemodynamics in mice sural nerves located in the hindlimb using *in vivo* two-photon laser scanning microscopy (TPLSM), based on techniques previously used to study perfusion across individual capillaries in the cerebral cortex in rats (Kleinfeld et al., 1998). Because peripheral nerves are prone to temperature changes (Dines et al., 1997), which occur in relation to the surgical exposure necessary for *in vivo* microscopy (Roche et al., 2019), we first applied the method to characterize the effects of local hindlimb temperature on hemodynamic recordings in the sural nerves of mice.

## Materials and Methods

### Animals

Male C57BL/6JBom mice (Taconic, Lille Skensved, Denmark) were acclimated to our animal facility for a minimum of 1 week. Mice were housed in groups at 22–24°C and approximately 55% air humidity with a 12:12 h light:dark cycle starting at 7 AM. Mice had access to water and chow *ad libitum* (Altromin, 1324 Rodent Diet, Brogaarden, Gentofte, Denmark) at all times.

Ten mice underwent *in vivo* imaging at the age of 15–19 weeks, weighing 27–37 g (mean ± SEM 31.1 ± 0.8 g). Additional five mice at the age 18–19 weeks, weighing 28–35 g (mean ± SEM, 30.2 ± 1.2 g) were used for perfusion with gelatinized India Ink and the following histology analyses.

### Histology

#### India Ink Perfusion

Mice were anesthetized with 5% isoflurane inhalation in atmospheric air followed by an intraperitoneal injection of sodium pentobarbital overdose. Immediately after cessation of spontaneous respiration, mice were placed under a heating lamp to keep the temperature at 37°C, the heart exposed by an incision through the ribcage, and a needle connected to a perfusion pump inserted into the left ventricle. A small hole was cut in the right ventricle and the mouse was then perfused with warm (37°C) heparinized PBS for 10 min at a flow rate of 4 ml/min using a peristaltic perfusion pump (Minipuls 3, Gilson, Villiers, France). This was followed by 5 min of perfusion with a warm (37°C) solution of 10% India Ink in deionized water with 2% gelatin (Xue et al., 2014) at a flow rate of 4 ml/min. The carcass was then placed in a sealed plastic bag and immersed in ice water for 30 min to solidify the gelatin. The skin on the lower hindlimb was removed and the sural nerve with its over- and under-laying muscles was dissected and embedded in a mold in optimal cutting temperature compound (QPath, VWR Inc., West Chester, PA, United States) and frozen on dry ice.

**FIGURE 1:**
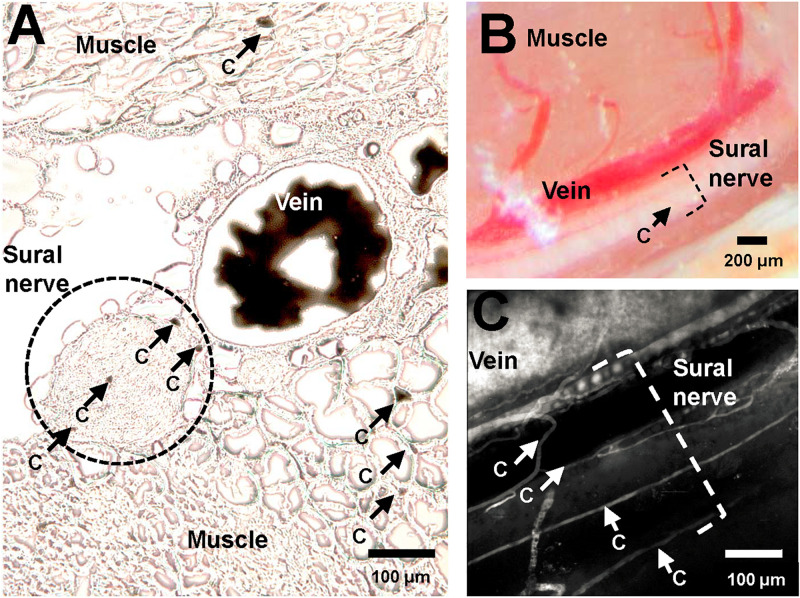
Vasculature of murine sural nerve. **(A)** Cross section of murine sural nerve and surrounding tissue at the approximate location of *in vivo* imaging. Vasculature visualized using perfusion with gelatinized India Ink. Frozen, unfixed 10 μm thick sections visualized under light microscope. Sural nerve highlighted by a dashed black circle. Some tissue capillaries in nerve and muscle are indicated by black arrows and letter “c”. **(B)** Murine sural nerve preparation visualized through surgical microscope after preparation of sural nerve window. Sural nerve highlighted by black dashed bracket. One small vessel, indicated by a black arrow and letter “c” can be seen crossing the nerve. **(C)** Maximum intensity projection image of a volumetric two-photon *in vivo* scan of murine sural nerve and its vasculature. Only vasculature is labeled with fluorescent dye, therefore it appears in white. The nerve is not labeled, therefore it is not visible, but its location is highlighted with a white dashed bracket. Sural nerve vessels where single file RBC passage has been observed indicated with white arrows and letter “c”.

Ten μm thick transversal sections of the lower limb muscles and nerve were cut using a cryostat (Cryostar NX70, Thermo Scientific, Waltham MA; United States) and immediately visualized under light microscope (Leica DM5000 B, Wetzlar, Germany). Images were captured and then analyzed using Fiji (Schindelin et al., 2012; Rueden et al., 2017). One section was analyzed for each mouse to determine the cross-sectional area of each nerve fiber, and the number of vessels within (endoneureal vessels) and directly adjacent to (epineureal vessels) the nerve fiber. Epineural vessels were subdivided divided into “large” and “small” adjacent vessels, as illustrated in Figures 1A,B, where “vein” is a “large” adjacent vessel, and the capillaries labeled “c” are small epineural vessels. Then, the total number of vessels per nerve fiber area were calculated, including the large and small vessels.

### *In vivo* Imaging

#### Surgical Preparation

On the day of *in vivo* imaging, each mouse was weighed and anesthetized by 5% isoflurane mixed with medical air supplemented with oxygen to a FiO_2_ of approximately 25%. Immediately following induction, the mouse was placed on a heating pad and its core body temperature maintained at 37°C with a thermostat connected to the mouse rectal probe (HB 101/2, Harvard Apparatus, Holliston, MA, United States). Isoflurane was maintained at 1.75–2% during surgery, and reduced to 1–1.25% during *in vivo* imaging protocol.

Following local hair removal and disinfection with 70% (v/v) alcohol, the trachea was exposed by a midline skin incision and blunt dissection through connective tissue and muscle. Following a small incision in the trachea just below the larynx, a 2.5 cm long, 0.86 mm inner diameter (ID) and 1.27 mm outer diameter (OD) PE-90 polyethylene tube (BD Intramedic, Clay Adams Brand, Sparks, MD, United States) was inserted into the trachea and secured by sutures. After the skin was sutured, the tube was connected to a mechanical ventilator (SAR-830/AP ventilator, CWE Inc., Ardmore, PA, United States) with ventilation flow set at 60 cc/min, individually adjusted ventilation rate, and 1:1 inspiration/expiration ratio. End-tidal CO_2_ levels in expired air were monitored using a micro-capnograph (Microcapstar, CWE Inc., Ardmore, PA, United States), which was calibrated prior to surgery using two point calibration using medical air and 5% CO_2_.

An inguinal skin incision was performed in the left hindlimb to cannulate the femoral artery and vein using 0.28 mm ID and 0.61 mm OD PE-10 polyethylene catheters (BD Intramedic, Clay Adams Brand, Sparks, MD, United States). The venous catheter was used for fluorescent dextran dye injection, and the arterial catheter for continuous blood pressure and heart rate monitoring (BP-1 system, WPI Inc., Sarasota, FL, United States). Arterial pressure, ventilation rate, and end-tidal CO_2_ were recorded and stored using 16/35 PowerLab data acquisition system (ADInstruments, Oxford, United Kingdom). Mean arterial pressure (MAP) and heart rate (HR) were calculated from the continuous blood pressure curves.

#### Nerve Window

**FIGURE 2:**
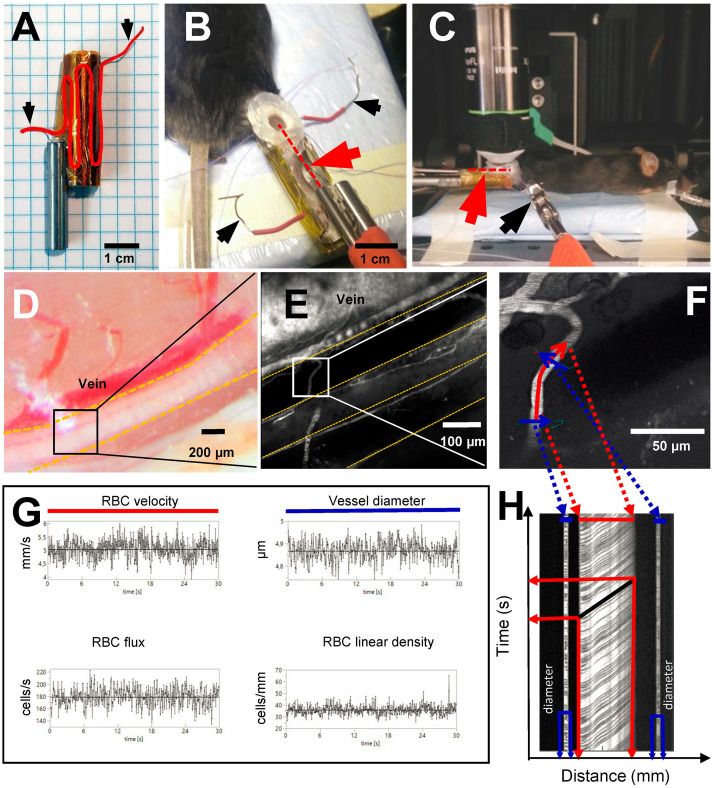
Experimental setup and line scan measurements. Black arrows show where power source is connected to resistance wire in order to warm up the leg holder. **(A)** Custom made leg holder with resistance wire highlighted in red; **(B)** Mouse right hindlimb fixed in the leg holder with an imaging window prepared over the sural neve. Red dotted line and red arrowhead indicate the location of the temperature probe inside of the preparation; **(C)** Anesthetized mouse in *in vivo two-photon* microscope ready for imaging. Red arrowhead and red dotted line show the location of temperature probe inside the preparation; **(D)** Sural nerve of the mouse highlighted by yellow dotted lines. Black square indicates imaging location for *in vivo two-photon* microscopy; **(E)** Vasculature of the sural nerve labeled with Texas red dextran. Sural nerve is highlighted by yellow dotted lines, but appears black as it is not labeled; vessels appear in white due to labeling. **(F)** Magnification of a microvessel of sural nerve, where line scan has been performed. Red line shows scan path along the vessel for measurement of RBCv, blue lines show scan path across the vessel for measurement of vessel diameter; **(G)** Typical measurements of RBCv, vessel diameter, RBC flux and RBC linear density acquired for 30 s from line scans; **(H)** Example of a line scan acquired for 30 s in a sural nerve microvessel. Black lines are individual red blood cell shadows. Vessel diameter can be estimated from the transversal part of the line scan (in blue), RBCv can be estimated from the axial part of the line scan (in red) by dividing distance traveled by the individual cell by the time of travel.

Following intubation and catheterization, the mouse was placed in a prone position and the right hindlimb was fixed in a custom leg holder. The holder was made from a 3.5 cm long hollow (8 mm ID, 10 mm OD) stainless steel tube with the top section removed (Figures 2A,B). The outside of the holder (Figure 2A) was covered with kapton tape and a 17.5 cm long piece of resistance wire (0.4 mm diameter, 3.9 ohm/m) was folded around it and secured using a second layer of kapton tape to ensure electrical insulation. The exposed ends of the resistance wire were connected to an adjustable linear power supply (GPS-2302, GW Instek, Taiwan) which was used to warm the fixed hindlimb to a desired temperature.

The right hindlimb was fixed in the holder using luting dental cement (GC Fuji PLUS, Leuven, Belgium). Following a popliteal incision, the semitendinous and posterior femoral biceps muscles were isolated by blunt dissection and retracted with sutures to expose the sural nerve without causing bleeding (Figures 2B,D). If any muscle bleeding occurred, hemostasis was secured by extra fine cauterizing forceps. The nerve was covered with a drop of low viscosity silicone (KWIK-SIL, World Precision Instruments, Sarasota, FL, United States) and a round 5 mm diameter coverslip. Dental cement was applied around the edge of the coverslip, fixing it to the leg holder and thus creating a stable sural nerve window (Figure 2B). Following these procedures, a 3 cm long, 0.33 mm hypodermic needle microprobe with a thermocouple (time constant 0.025 s, MT-29/3, Physitemp Instruments Inc, Clifton NJ, United States) was inserted into the leg, proximad and parallel to the nerve, and advanced into the nerve window beneath the imaging plane (Red dotted line in Figure 2B). The probe was connected to the BAT-12 microprobe thermometer (Physitemp Instruments Inc., Clifton NJ, United States) and temperature was recorded using Power Lab data acquisition system.

After the completion of surgical preparation, the mouse was positioned under the objective of the two-photon laser microscope (Figure 2C).

Nerve perfusion was studied at peripheral temperatures of 37, 32, and 28°C, as recorded in the hindlimb of the anesthetized mice using the temperature needle microprobe. These temperatures were chosen, as they typically occur in the hindlimb before surgical exposure, after surgical exposure and window preparation, or after positioning for imaging using water immersion objective, respectively.

#### *In vivo* Imaging Protocol

Nerve perfusion measurements were conducted using a Prairie Ultima-IV *In vivo* Laser Scanning Microscope (Brucker Corporation, Billerica, MA, United States) equipped with an Olympus water immersion 20X objective with 1.0 numerical aperture and 2.0 mm working distance, with and *x*–*y* resolution of 0.21 μm and depth resolution of 0.81 μm. To image nerve vasculature, plasma was labeled by injecting 150 μL of 0.5% Texas-red dextran solution in sterile 0.9% saline (70,000 MW, 5 mg/mL, Thermo Fisher Scientific) through the femoral vein catheter. The dye was excited using a laser light wavelength of 900 nm, and fluorescence detected by a GaAsP photomultiplier (Hamamatsu, H7422-40) using a 660/40 nm-emission filter. Using this approach, the blood plasma fraction appears bright in the acquired images (Figure 2E), while unlabeled RBC appear as dark shadows (Figures 2F,H).

Throughout imaging, mouse core body temperature was maintained at 37°C, while hindlimb temperature was adjusted to 28, 32, and 37°C in a randomized order for nerve perfusion measurements. Accordingly, at the start of the *in vivo* imaging protocol, the temperature was set to one of the three temperatures, and nerve perfusion was measured within 3 min after the temperature had stabilized at the desired level. The time needed for limb temperature to stabilize never exceeded 12 min.

Red blood cell velocity (RBCv, mm/s), flux (RBC/s), linear density (LD, RBC/mm), and vessel diameter (μm) were measured in sural nerve microvessels where single file RBC passages were observed, avoiding vessels with stalled or no flow at the initial selection. Accordingly, in each animal, the selected vessels were scanned at all three temperatures when possible. In some instances, microscopic shifts in tissue position or technical issues related to TPLSM, prevented data acquisition during all three conditions. RBC dynamics were measured as previously described (Gutierrez-Jimenez et al., 2016). Briefly, line scans were planned using the “freehand line” function of the microscope. The line scan path was prescribed both along and across the axis of each vessel. Figure 2F shows a typical example of such a line for the line scan. The scan data acquired along the midline of the vessel, indicated by the red line in Figure 2F, was used to estimate RBCv, flux, and LD. The scan data acquired along the line perpendicular to the vessel, indicated by the blue lines in Figure 2F, was used to estimate vessel diameter, and to provide an additional estimate of RBC flux and LD. The signal along each line scan was continuously recorded for 30 s at each of the temperatures.

Once the line scans had been performed at all temperatures, an angiogram of the nerve’s vasculature was acquired by recording a stack of two dimensional images in the z-direction, as deep as the preparation allowed.

Immediately after imaging, the mice were euthanized by sodium pentobarbital overdose (Exagon vet., Richter Pharma, Wels, Austria).

#### Image Processing

Vessel diameter, RBCv, flux and linear density were estimated from the line scans using software developed in-house for Matlab (R2016b, Mathworks Inc.) as previously described (Drew et al., 2010; Gutierrez-Jimenez et al., 2016).

The line scans acquired during the 30 s were stacked to create a two dimensional raw image with time on *Y*-axis and distance on *X*-axis (Figure 2H). In these images dark, angled streaks appear on the axial portion of the line scan (indicated in red in Figures 2F,H). The angle of each streak shows displacement of the individual RBC on both time and distance axes thus resulting in RBCv. These angles were determined automatically using Radon transform algorithm. Velocity estimates with signal to noise ratio below three were excluded from the analyses (Drew et al., 2010).

Vessel diameter was estimated from the cross sectional part of the line scan (indicated in blue in Figures 2F,H) as a full width at half maximum, assuming that the cross section represents the full diameter of the vessel. This was only done in the “light” portions of the cross sectional scan where no RBCs were present. RBC flux was determined utilizing temporal intensity changes in the signal, indicating presence or absence of RBC both from axial and cross sectional parts of the line scans. The intensity variation was analyzed using cluster analysis to determine presence of RBC in the vessel at the given time and RBC flux (cells per second) was calculated. RBC linear density was calculated as a ratio between RBCv and flux.

All estimates were derived using a sliding window approach. Accordingly, starting from time zero of each 30 s line scanning data set, each parameter (RBCv, Flux, LD, and capillary diameter) was estimated from data within a smaller time window with a specified duration. By gradually moving this time window by specified time-shifts (“sliding”) for the length of the 30 s scan, the dynamics of each parameter could be characterized by their time-course, as determined within time windows with a certain duration and overlap (Supplementary Figure 1). We tested a number of window durations and time shifts (overlap) prior to the data analysis (Supplementary Figure 1) to determine the most appropriate time resolution. Time windows of 100 ms duration and 50 ms time shift proved to provide the most reliable estimates, and sufficiently high temporal resolution to allow further power spectral density analyses (Supplementary Figure 1). Time series of all estimates were filtered for outliers, which were defined as data points more than five median absolute deviations away from the median. Identified outlier points were excluded and interpolated with the nearest within-range values.

For temperature effects, we report averaged parameter values over the entire 30 s periods.

#### Blood Flow Oscillations

To determine whether our RBCv measurements were sufficiently sensitive to detect subtle oscillations in blood flow across different timescales (Stefanovska et al., 1999; Smirni et al., 2019), we subjected RBCv time series to power spectrum analysis using Matlab’s Welch’s power spectral density estimation function. Such oscillations are observed in peripheral vessels in mice and humans and they have been ascribed to specific cellular and physiological sources according to their characteristic frequency ranges (Stefanovska et al., 1999; Smirni et al., 2019). Based on the literature, we pre-defined frequency bands to include oscillations characteristic to their sources: 0–0.02 Hz (endothelial), 0.02–0.05 Hz (neurogenic), 0.05–0.15 Hz (myogenic), 0.15–3 Hz (respiration), and 3–10 Hz (heart rate). Of these, the latter two were expected to include the respiration and heart rates characteristic of our experiments.

### Statistical Analyses

Data was analyzed by fitting a restricted maximum likelihood model with temperature and MAP as fixed effects, and individual vessels as a random effects using JMP 14.0 (SAS Institute Inc., Cary, NC, United States). MAP was included in the model as a covariate, therefore, when it did not have a significant effect on the parameter in question, it was removed from the model, and the reduced model fitted again. If temperature had a statistically significant effect we used the least squared means differences Tukey test as a *post hoc* test.

For outcomes that were estimated in duplicate (diameter) or triplicate (flux and linear density) per vessel, all measurements were included in the statistical analyses, by nesting them within each individual vessel. For flux and linear density estimates we also included the “estimation segment” (axial or transversal) as a covariate in the statistical model.

To evaluate whether vessel diameter had an effect on RBC velocity, flux or linear density a standard least squares model was fitted using JMP 14.0 with vessel diameter and temperature as model effects.

Data was visualized using Prism 8.3 (GraphPad Software Inc., San Diego, CA, United States).

All data presented as means ± standard error of mean (SEM), unless otherwise indicated.

## Results

### Nerve Vasculature

Figure 1A shows a typical example of a sural nerve cross section following perfusion with gelatinized India Ink. The sural nerve, located between muscle tissue, is highlighted by the black dotted line. Few endoneureal capillaries and several small epineureal vessels (marked with “c”) can be observed both in nerve sections stained with gelatinized India Ink (Figure 1A) and in angiograms recorded *in vivo* using TPLSM (Figure 1C), but are barely discernible using the surgical microscope (Figure 1B). Across the five samples analyzed, there were between 0 and 3 endoneureal capillaries, and between 0 and 5 epineureal small vessels per sample. Typically, the sural nerve was comprised of one to three nerve bundles, with a mean diameter of 167 μm (SEM ± 34 μm) and cross section area of 0.026 ± 0.009 mm^2^. The nerve always traveled in parallel with a large vessel of similar caliber with a mean diameter of 114 μm (SEM ± 27 μm). On average, the total number of vessels associated with the nerve (including the large vessels) resulted in 330 ± 97 vessels per mm^2^ of the nerve. Omitting the large vessels from the estimate to obtain only microvascular vessel density resulted in 237 ± 80 vessels per mm^2^, while including only the endoneureal vessels resulted in 52 ± 25 vessels per mm^2^.

**FIGURE 3:**
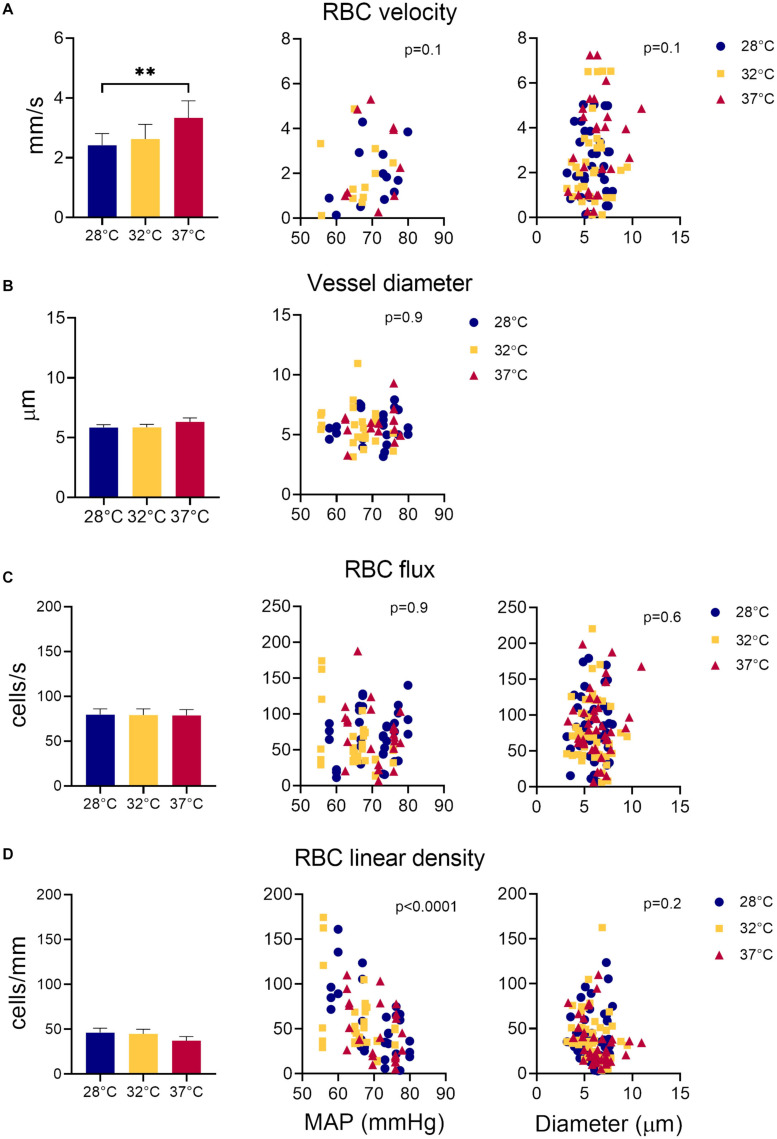
Measurements of sural nerve blood flow. Effect of temperature, MAP, and vessel diameter on **(A)** RBC velocity; **(B)** vessel diameter; **(C)** RBC flux, and **(D)** RBC linear density. Values shown as mean ± standard error. Values compared by fitting a restricted maximum likelihood model with temperature as a fixed effect and MAP as fixed covariate in the model, and individual vessels as random effect. If MAP did not have a significant effect on the parameter in question, it was removed from the model, and the model fitted again. If temperature had a statistically significant effect, as a *post hoc* test, we used the least squared means differences Tukey test. Effect of vessel diameter and temperature on RBCv, flux and linear density estimated by fitting a least square means model with vessel diameter and temperature as model effects. **p < 0.01, *p < 0.05.

The average sural nerve vessel diameter, measured during *in vivo* microscopy was 6 μm, ranging from 3.14 to 10.96 μm (Figure 3B). Note that only vessels with single file RBC passage were imaged, therefore larger vessels of the sural nerves were not included in this estimate. Hindlimb temperature or MAP did not affect vessel diameter (Figure 3B).

### Nerve Perfusion

Overall, RBCv in sural nerve vessels ranged broadly from 0.12 – 7.25 mm/s with a mean velocity of 2.77 mm/s. Figure 3A shows an increase in RBCv with increasing hindlimb temperatures (*p* = 0.002). Compared to 28°C, RBCv was 14% higher at 32°C and 33% higher at 37°C. However, the difference was only statistically significant comparing RBCv at 28°C and 37°C where the difference (Δ) was 0.88 with confidence interval (CI) [0.33; 1.44] (*p* = 0.0015), but not between 28°C and 32°C (Δ = 0.48, CI [−0.02; 0.98], *p* = 0.06) or between 32°C and 37°C (Δ = 0.40, CI [−0.16; 0.96] *p* = 0.19). RBCv was not affected by MAP.

Neither RBC flux (Figure 3C) nor LD (Figure 3D) were affected by hindlimb temperature. Increased MAP lead to a decrease in RBC LD (Figure 3D, estimate = −2.66, *p* < 0.0001) but had no effect on RBC flux (Figure 3C). Estimates of both RBC flux and LD were significantly higher when derived from the transversal part of the line scan, compared to those derived from the axial part of the line scan (Flux: *p* < 0.0001, Estimate = 15.4; LD: *p* < 0.002, Estimate = 6.16).

Vessel diameters did not have a significant effect on RBCv, flux or LD.

### RBCv Oscillations

**FIGURE 4:**
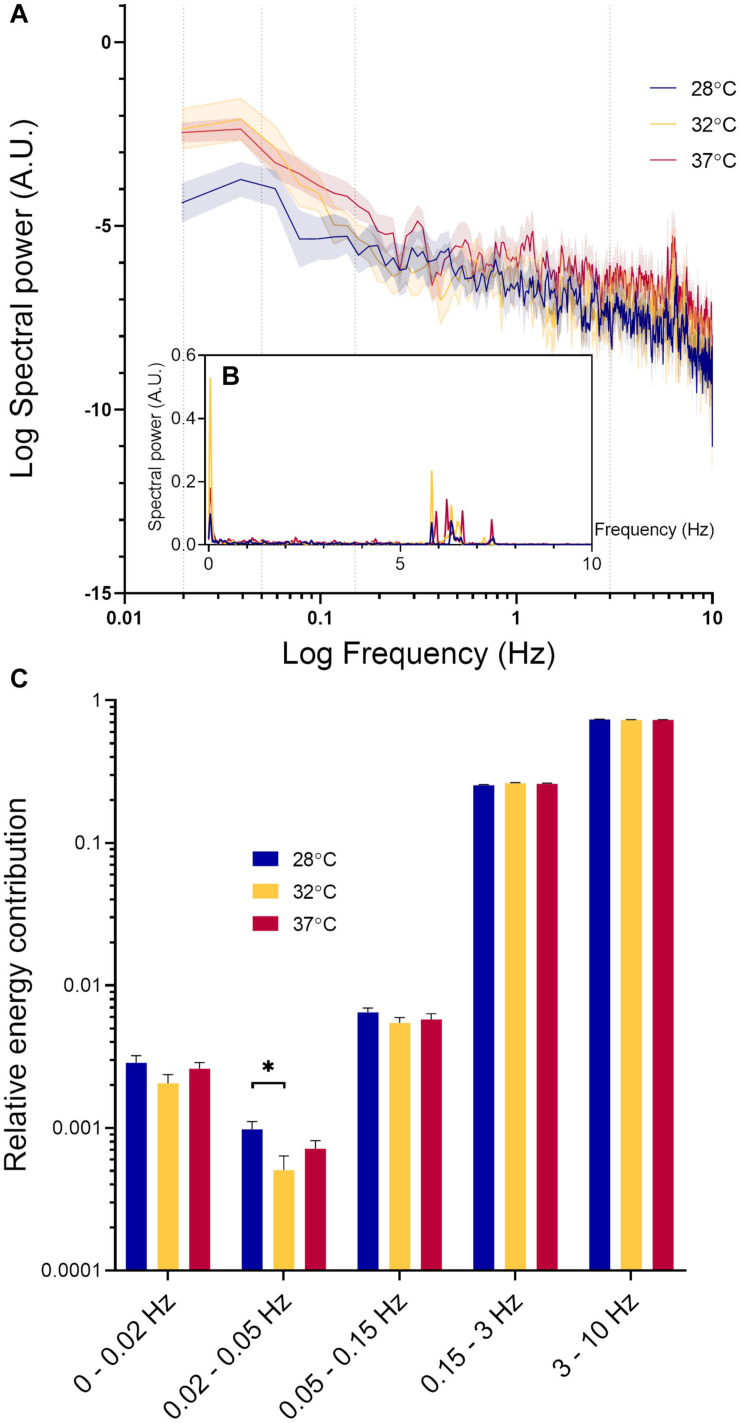
Spectral power density analysis of RBCv signal. **(A)** Distribution of spectral power density (Mean ± standard error) at three different hindlimb temperatures on logarithmic scale. Values at 0 Hz are absent on the graph due to the logarithmic scale; **(B)** Distribution of spectral power density (Mean ± standard error) at three different hindlimb temperatures; **(C)** Relative energy contribution by defined frequency bands: 0–0.02, 0.02–0.05, 0.05–0.15, 0.15–3, and 3–10 Hz. Each band compared by fitting a restricted maximum likelihood model with temperature and MAP as fixed effects in the model, and individual vessels as random effect. If MAP did not have a significant effect on the parameter in question, it was removed from the model, and the model fitted again. If temperature had a statistically significant effect, as a *post hoc* test, we used the least squared means differences Tukey test *p < 0.05.

Figure 2G shows a typical example of RBCv signal acquired for 30 s. From this recording, it is possible to observe both rapid fluctuations in RBCv, as well as slower oscillations around the mean, as indicated by the black horizontal line. To characterize this variability in RBCv, we performed a power spectral density analysis on each of the acquired time series and compared the outcomes at different temperatures. Figure 4A shows how the power of RBCv oscillations is distributed by frequencies. Pronounced peaks in spectral power appear at frequencies between 5.5–8.8 Hz (Figure 4 and Supplementary Figure 2). These peaks correspond well to the heart rate measured by arterial catheterization, which averaged at 6.7 ± 0.4 Hz. During imaging, mice were mechanically ventilated at an average frequency of 1.6 ± 0.05 Hz, however, no clear peaks appear at this frequency after power spectral density analysis (Figure 4 and Supplementary Figure 2). Another pronounced peak appears at low frequencies (0–0.15 Hz) at all three hindlimb temperatures (Figure 4).

In Figure 4C, the spectral power density of RCBv oscillations was normalized and grouped according to the five predefined frequency bands. The high frequency oscillations (3–10 HZ) had the highest relative power contribution, followed by oscillations at the lower frequencies (0.15–3 Hz). Spectral power within these intervals was not significantly affected by MAP or temperature. The relative power contribution from the low frequency oscillations (0–0.15 Hz) was much lower than that by the high frequency oscillations (0.15–10 Hz) at all temperatures. The oscillations within frequency range 0.02–0.05 Hz were significantly affected by temperature (*p* = 0.03). Pairwise comparison revealed a statistically significant difference between 28°C and 32°C (Δ = 0.00046, CI [0.000058; 0.00087], *p* = 0.02), but not between other pairs. RBCv oscillations within the lowest frequency band 0–0.02 Hz increased significantly with increasing MAP (Estimate = 0.00007, *p* < 0.05), but not by temperature. Finally, oscillations in RBCv between 0.05–0.15 Hz were not affected by MAP or temperature.

## Discussion

This study presents a nerve window technique, whereby the hindlimb of a mouse is fixed and the sural nerve exposed to allow for *in vivo* TPLSM studies and careful hindlimb temperature control. This preparation is sufficiently stable to allow *in vivo* observations of the nerve’s microcirculation, including line-scanning to detect the passage of individual RBCs through capillary-sized nerve microvessels. The RBCv measurements were sufficiently sensitive and robust to detect subtle flow oscillations over time scales from 0.1 to tens of seconds. Our measurements show that RBCv in murine distal sural nerves increases with temperature, underscoring the importance of maintaining hindlimb temperature constant at the physiologically relevant level of 37°C. Using TPLSM, we observed that the sural nerve receives its blood supply through a few small vessels, located on the surface of the nerve as well as within the nerve. Using sural nerve sections labeled with gelatinized India Ink, we were able to verify that *in vivo* measurements were comprehensive and representative of the whole vasculature of the nerve, as the number and size of sural nerve vessels observed in sections corresponded well with those we observed during *in vivo* scans.

The diameter of vessels within which we recorded RBC dynamics using TPLSM is similar to that described in brain tissue of mice and rats (Kleinfeld et al., 1998; Stefanovic et al., 2008; Gutierrez-Jimenez et al., 2016; Dorr et al., 2017; Anzabi et al., 2019) and in rat tibial nerves (Sakita et al., 2014, 2016). This is in contrast to a detailed morphological study of the more proximal, sciatic nerve vasculature in rats and mice, where the smallest nerve vessel sizes were between 8 and 9 μm in diameter, larger than muscle tissue microvessels (ca. 5 μm in diameter) (Bell and Weddell, 1984). These differences might be due to the different methods used to measure vessel diameters. *In vivo* microscopy, used in our study and in the five studies of the brain (Kleinfeld et al., 1998; Stefanovic et al., 2008; Gutierrez-Jimenez et al., 2016; Dorr et al., 2017; Anzabi et al., 2019), reveals vessel diameters without artifacts induced by tissue fixation, but also selects vessels with single file RBC passage, possibly underestimating mean tissue microvessel diameter. In the study of the rat tibial nerve, whole vasculature was perfused with gelatinized contrast and sections were studied (Sakita et al., 2014, 2016). In this way, the inclusion of vessels of all diameters (i.e., also those larger than the vessels with single file RBC passage) may lead to higher vessel diameter estimates. How the fixation artifact may change diameter estimates compared to vessel diameters *in vivo* is uncertain due to the finite thickness of the vessel wall and its constituents (basal membrane, endothelial cells, glycocalyx layer) and possible varying effects of fixation on them. Hence, the difference between our findings and those reported by Bell and Weddell (1984) might be explained by tissue fixation. Additionally, species used, location of the nerve, and even the age of the animal can affect vessel diameter, as shown by (Sakita et al., 2014, 2016) where tibial nerve capillary diameter was reduced with age in rats.

When only the endoneureal vessels are included in the estimation of sural nerve vessel density in mice our results show a density of 52 vessels per mm^2^. This is comparable to studies in human sural nerves (Mohseni et al., 2017; Kan et al., 2018) and in rat sciatic nerves where endoneureal vessel density ranges between 49 and 98 vessels per mm^2^, respectively. Yet these values are well below those reported for brain and muscle tissue, where vessel density can be as high as 375 vessels/mm^2^ in the mouse (Li et al., 2008) and 244 vessels/mm^2^ in rat brain and up to 500 vessels/mm^2^ in mouse muscle tissue (Bell and Weddell, 1984). When we included all the vessels associated with the sural nerve in the vessel density calculation (small endoneureal vessels, small vessels on the nerve’s surface and the large adjacent vessels), vessel density approached that observed in brain and muscle tissue at ca. 330 vessels/mm^2^. As arterioles have previously been reported to play a role in oxygen diffusion in the muscle (Ellsworth and Pittman, 1990) and in the brain (Sakadžić et al., 2014), it is reasonable to include them in sural nerve vascular density estimates, as they are likely to contribute to oxygenation of the sural nerve. In addition, in such small nerve fibers as murine sural nerve, the small “epineureal” vessels are likely to act as nutritive vessels, due to absence of thick diffusive barriers, such as epineurial connective tissue.

RBCv in sural nerves in mice was several fold higher than that previously reported in mice and rat brain capillaries using TPLSM where RBCv values ranged between 0.4 mm/s to 1.4 mm/s in mice (Gutierrez-Jimenez et al., 2016; Gutiérrez-Jiménez et al., 2017) and 0.77 mm/s in rats (Kleinfeld et al., 1998) under varying anesthetic protocols. In fact, RBCv in sural nerve was more similar to cortical arteriolar RBCv of 2.4 mm/s measured in mice under a matching anesthetic protocol (Dorr et al., 2017). Cortical arterioles, however, had much larger vessel diameters (ca. 12 μm) compared to the vessels of the sural nerve (6 μm). RBCv in cortical arterioles was measured using a water immersion objective, without additional control of surface temperature of the brain, and, as has been shown by (Roche et al., 2019), this not only significantly reduces cortical temperature, but also RBCv and flux both in awake and anesthetized mice. Maintaining cortical temperature at 37°C in awake mice or at 35–36°C in anesthetized mice using either a dry air objective, or a warmed water immersion objective leads to significantly higher RBCv and flux measurements in cortical capillaries (Roche et al., 2019). This corresponds well with our findings of increased RBCv in sural nerve vessels with increasing hindlimb temperatures (Figure 3A), as we found a non-significant 15% increase in RBCv from 32°C to 37°C and a greater, statistically significant 33% increase in RBCv from 28°C to 37°C. Alongside the study by (Roche et al., 2019), our results highlight the importance of careful control of experimental variables on primary experimental outcomes, such as changes in tissue temperature due to imaging with a water immersion objective. As cortical arteriolar RBCv of 2.4 mm/s was measured using a water immersion objective, without specifically controlling brain’s surface temperature (Dorr et al., 2017), it is likely that the surface of the brain during these measurements was similar to that measured by (Roche et al., 2019) under similar conditions and was ca. 32°C. Also in the sural nerve vessels at 32°C and 28°C RBCv was 2.6 and 2.4 mm/s, respectively. Therefore, RBCv in sural nerves is more similar to RBCv in cortical arterioles and not capillaries, in spite of diameter differences.

It is well established that blood flow increases in response to local heating in skin, muscle and brain (Heinonen et al., 2011; Wang et al., 2014). In cerebral circulation blood flow acts as a heat sink, removing excess heat from the brain parenchyma (Wang et al., 2014). In the skin, which acts to dissipate the heat from the body, increase in the blood flow has been ascribed to a combination of neural regulation and local nitric oxide release (Minson et al., 2001). In the muscle, both nitric oxide release and elevation of tissue oxygen consumption at increased temperatures (Q_10_effect) are thought to have an effect on local blood flow increase (Heinonen et al., 2011). RBC flux in sural nerves (79 cells/s) was comparable to that previously reported in murine brain capillaries under isoflurane anesthesia [62 cells/s (Gutierrez-Jimenez et al., 2016)]. However, unlike in the brain where increasing surface temperature elevated RBC flux (Roche et al., 2019), warming of the hindlimb did not increase RBC flux in the sural nerve (Figure 3C). The absence of temperature effect on RBC flux in our study is also in contrast to previous measurements of sciatic nerve blood flow in rats measured using LDF, where nerve blood flow was *lower* at higher hindlimb temperatures (Dines et al., 1997, 1999). In these studies the decrease in nerve blood flow was paralleled by an increase in blood flow to the muscle (Dines et al., 1997, 1999). The absence of RBC flux changes in response to warming in sural nerve in our study points to a tight local regulation of blood flow at the microvascular level. In peripheral nerves microvascular innervation and local endothelial signaling play an important role in maintaining the vascular tone (Zochodne, 2018) and power spectral density analysis of RBCv signal indicates that both mechanisms might be at play in the microvasculature of the sural nerve (Figure 4). Power spectral density analysis showed that RBCv oscillations between 0.02 and 0.05 Hz, potentially corresponding to neurogenic regulation (Smirni et al., 2019), were reduced at 32°C compared to 28°C. Oscillations at the very low frequencies (0–0.02 Hz), potentially corresponding to the endothelial involvement and nitric oxide release (Stefanovska et al., 1999; Smirni et al., 2019), appeared more pronounced at 32°C and 37°C than at 28°C but were not statistically different, however, were significantly increased by increasing MAP. Even though the relatively short recoding lengths of 30 s limit the strength of conclusions that can be derived from the changes in oscillations in RBCv signal, the analysis shows that local blood flow control in the nerve is affected by changing temperatures and by MAP. Further studies designed to target endothelial, neurogenic and myogenic regulatory mechanisms in sural nerve vasculature are necessary to elucidate their role in local nerve blood flow regulation at capillary level. In previous studies MAP increased sciatic nerve blood flow in rats (Low and Tuck, 1984), however, we did not observe a statistically significant effect of MAP on RBCv or RBC flux. There was, however, a significant effect of MAP on RBC linear density (Figure 3D) with linear density decreasing with increasing MAP. The absence of effect of MAP on RBCv and RBC flux in our study is also likely due to the small vessels examined in our study.

### Limitations to the Study

Anesthesia inadvertently and inevitably affects animal physiology, thus making studies of blood flow under any anesthetic regime inherently challenging. Without consideration for the local temperature, a wide range of RBCv values has been measured in brain capillaries of mice under different anesthetics. Highest values in C57 male mice are reported under isoflurane anesthesia (1.4 mm/s) (Gutierrez-Jimenez et al., 2016), with much lower values under α-chloralose anesthesia (0.4 mm/s) (Gutiérrez-Jiménez et al., 2017). These values differ from those recoded in brains of awake mice first by Drew et al. (2010) where values ranged between 0.1–2 mm/s and by Roche et al. (2019) with RBCv at 0.75 mm/s. But all are lower than RBCv measured in the vessels of the sural nerve (from 2.4 mm/s at 28°C to 3.3 mm/s at 37°C) under isoflurane anesthesia. It is possible that isoflurane anesthesia in this experiment caused sural nerve RBCv to increase, such as it has been observed in the brain (1.4 mm/s on isoflurane vs 0.75 mm/s awake), where it is attributed to vasodilatory effects of isoflurane (Matta et al., 1999). The vasodilatory effect might have also lead to an overestimation of vessel diameters. However, even in that case, capillary RBCv in mouse sural nerves is higher than in the brain, while diameters of vessels with single file RBC passages are comparable to those in the brain (Dorr et al., 2017; Anzabi et al., 2019). Although hemodynamic measurements in the brain tissue of awake mice are becoming more common (Roche et al., 2019), preparation and fixation of the hindlimb in awake mice is currently not done.

Another challenge is the terminal nature of the current study protocol. It is not possible to study how blood flow changes over the course of time in the same mouse through e.g., disease progression. Potentially, this might be amended by optimizing the surgical preparation of the sural nerve window, and enabling the mouse to be recovered after imaging to allow for repeated imaging over time. However, this might be faced with the challenge of the repeated surgery, which might lead to surrounding tissue damage, and thereby affect study results.

## Conclusion

The novel peripheral nerve window technique presented here is suitable for detailed investigation of sural nerve hemodynamics in mice. Moreover, our results show that *in vivo* TPLSM is a fitting approach for hemodynamic studies in sural nerves. In such small and distal peripheral nerve fibers, counting individual RBC passages and measuring RBC velocity in individual small vessels provides a comprehensive assessment of local hemodynamics. In future studies combination of this method with calcium imaging (Fontaine et al., 2018) or use of oxygen sensitive dyes (Sakadžić et al., 2014) will enable us to study how nerve’s activity and metabolism are supported by perfusion in health and disease. Furthermore, this method will also enable us to study how pharmacological or physiological challenges affect local hemodynamics in the nerve. Our results have also shown that during these studies it is important to control and report the exact temperature of the exposed hindlimb of the mouse, due to its effects on estimates of RBCv.

## Data Availability Statement

The raw data supporting the conclusions of this article will be made available by the authors, without undue reservation.

## Ethics Statement

The study was reviewed and approved by the Danish Animal Experiments Inspectorate, animal experimentation permit #2016-15-0201-00966.

## Author Contributions

AD and LØ conceived the study. AD performed the experiments, drafted the manuscript, and designed the figures. AD and PR processed and analyzed the data. PR developed methods for the data processing and analysis. All authors interpreted the results and read, made critical revisions to, and approved the final version of the manuscript.

## Conflict of Interest

The authors declare that the research was conducted in the absence of any commercial or financial relationships that could be construed as a potential conflict of interest.

## Supplementary Material

Supplementary Figure 1 RBCv image analyses and windowing. Vessel diameter, RBCv, flux and linear density were estimated from the line scans using a sliding window approach to provide time series of these estimates (Drew et al., 2010). Overlapping temporal windows are positioned over the 2D line scan images, and diameter, velocity, flux, and linear density are estimated from line scan data within each window. The temporal resolution and noise of the resulting time series depend on window size and window shift. Smaller window sizes increase the temporal resolution of the estimate, but also increase standard deviation of the estimate for the whole period of estimation. We tested several combinations of sizes and temporal shifts of the windows to establish how that affects the signal of interest (averages across the temporal sequence) **(A–E)**. Average RBCv **(F)** is not changed by the size and shift of the estimation window, until the window size becomes too small (in this case **(E)**: 10 ms window with 5 ms overlap). All estimates acquired from imaging were filtered for outliers defined as more than five median absolute deviations away from the median. Identified outlier points were replaced by interpolated values estimated from the nearest within-range values. The mean ± SD of the raw or unfiltered values are plotted in **(F)** as black symbols, and filtered values as open symbols.

Supplementary Figure 2 Example of spectral power density analysis of single velocity time series, estimated using a temporal window of 100 ms and a window shift of 50 ms (Supplementary Figure 1). A distinct density peak is observed at the measured hear rate. Frequency of measured ventilation is also indicated, but not easily discernible from the trace.
