# Supplementary figures and images for: Sural Nerve Perfusion in Mice
Source: Front Neurosci. 2020 Dec 10;14:579373. doi: 10.3389/fnins.2020.579373 (PMC7758475; doi:10.3389/fnins.2020.579373)

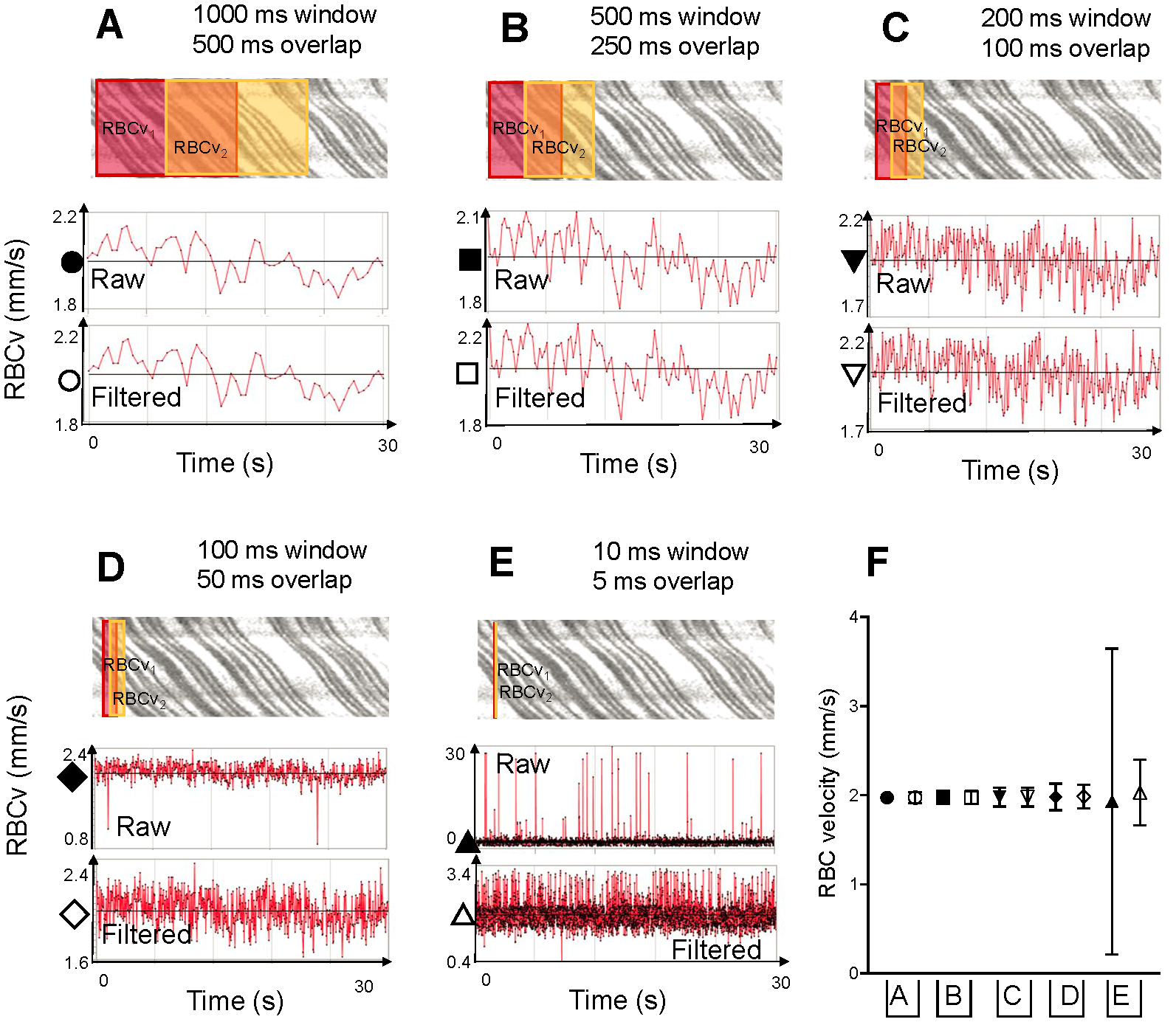

Supplement: Supplementary Figure 1 — RBCv image analyses and windowing. Vessel diameter, RBCv, flux and linear density were estimated from the line scans using a sliding window approach to provide time series of these estimates (Drew et al., 2010). Overlapping temporal windows are positioned over the 2D line scan images, and diameter, velocity, flux, and linear density are estimated from line scan data within each window. The temporal resolution and noise of the resulting time series depend on window size and window shift. Smaller window sizes increase the temporal resolution of the estimate, but also increase standard deviation of the estimate for the whole period of estimation. We tested several combinations of sizes and temporal shifts of the windows to establish how that affects the signal of interest (averages across the temporal sequence) (A–E). Average RBCv (F) is not changed by the size and shift of the estimation window, until the window size becomes too small (in this case (E): 10 ms window with 5 ms overlap). All estimates acquired from imaging were filtered for outliers defined as more than five median absolute deviations away from the median. Identified outlier points were replaced by interpolated values estimated from the nearest within-range values. The mean ± SD of the raw or unfiltered values are plotted in (F) as black symbols, and filtered values as open symbols. [file Image_1.TIF]

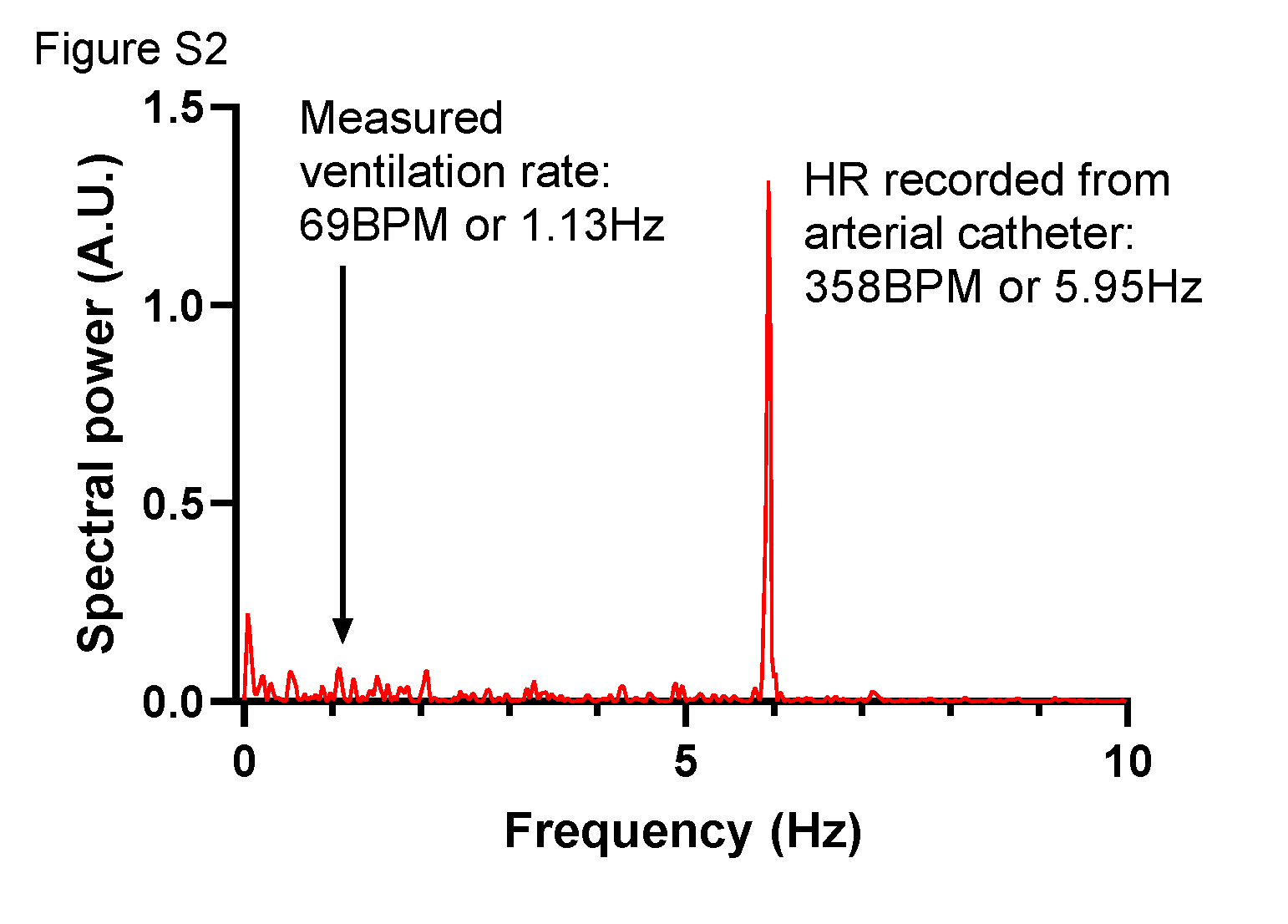

Supplement: Supplementary Figure 2 — Example of spectral power density analysis of single velocity time series, estimated using a temporal window of 100 ms and a window shift of 50 ms (Supplementary Figure 1). A distinct density peak is observed at the measured hear rate. Frequency of measured ventilation is also indicated, but not easily discernible from the trace. [file Image_2.TIF]
